# Numerical Fatigue Analysis of Dissimilar Lap Joints Fabricated by Dimple Spot Welding for Automotive Application

**DOI:** 10.3390/ma18030627

**Published:** 2025-01-30

**Authors:** Paolo Livieri, Michele Bortolan

**Affiliations:** Department of Engineering, University of Ferrara, via Saragat 1, 44121 Ferrara, Italy; michele.bortolan@unife.it

**Keywords:** fatigue, welded joints, implicit gradient, dimple spot welding

## Abstract

This paper presents a numerical analysis of dimple spot welding (DSW) as an innovative joining technique for dissimilar materials, namely steel and aluminium alloys. Employing a finite element (FE) model, the study simulates the fatigue performance of DSW joints, considering crucial factors such as contact friction and cyclic loading conditions. While various numerical models are proposed, the simulation incorporating friction and fatigue loading appears to offer the highest accuracy. The research highlights that the fatigue behaviour of DSW joints can be effectively investigated through the non-local theory of the implicit gradient approach by utilising the fatigue curve of arc-welded structures composed of steel or aluminium alloys. Specifically, simulations incorporating friction and fatigue loading demonstrate that the steel spot weld does not represent the weakest point within the joints.

## 1. Introduction

The automotive industry is increasingly focused on developing lightweight vehicle structures to enhance fuel efficiency and reduce emissions. This shift has necessitated the exploration of alternative joining technologies, particularly for lightweight metals like aluminium alloys, which are gaining prominence in modern vehicle designs. Traditional methods, such as spot welding [1,2,3], are used for joining steel body panels, but their application to aluminium has proven challenging. Aluminium’s high thermal conductivity, low melting range, and tendency to form an oxide layer complicate the spot welding process, often resulting in suboptimal joint integrity and reduced lifespan of welding electrodes [4]. To address these challenges, several alternative joining methods have been considered, including adhesive bonding [5], riveting [6], and clinching [7,8,9,10]. Among these, self-piercing riveting (SPR) has emerged as a promising technology for joining aluminium body panels. SPR operates as a cold forming process, allowing a semi-tubular rivet to be pressed into two sheets of material without the need for pre-drilled holes or precise alignment, thus simplifying assembly and enhancing the potential for joining dissimilar materials [11].

Recent studies have assessed the mechanical performance of SPR joints, revealing a complex interplay between static and fatigue strengths. For instance, while spot-welded joints often demonstrate higher static strength, SPR joints exhibit superior fatigue resistance. Research indicates that at 10^6^ cycles, the fatigue strength of SPR joints can be twice that of their spot-welded counterparts [12,13]. This distinction makes SPR joints particularly suitable for applications subjected to cyclical loading.

The effectiveness of an SPR joint hinges on the quality of the mechanical interlock formed during the riveting process. Different factors influence this interlock, including die geometry, sheet thickness, rivet properties, and surface coatings [14]. Studies have shown that modifying die dimensions can significantly affect rivet flaring and joint strength. Similarly, the length and diameter of the rivet play crucial roles; longer and thicker rivets tend to absorb more energy, enhancing joint robustness [15].

Another critical aspect influencing joint strength is specimen configuration. Research demonstrates that joint strength varies significantly based on whether the configuration is optimised for shear or peel tests, with shear configurations generally exhibiting higher strength [16]. The design of the joint itself is also pivotal; evidence suggests that double-riveted joints do not inherently surpass the performance of single-riveted joints when normalised for stress, emphasising the importance of optimal rivet placement and spacing [17].

Despite the advantages of SPR, the diverse combinations of material grades and thicknesses present challenges in determining optimal rivet and die configurations. The need for tailored processes for numerous combinations increases complexity and cost in production [18].

The mechanical behaviour of SPR joints is further complicated by the loading conditions they face in service. Studies have highlighted how rivet parameters, sheet thickness, and edge distance significantly affect both static and fatigue performance. For instance, increasing edge distance can reduce joint distortion and enhance strength [19]. In summary, while the self-piercing riveting process offers a viable alternative to traditional joining methods for aluminium and dissimilar materials, ongoing research is essential needed to fully understand and optimise its mechanical performance. The exploration of SPR’s capabilities and limitations is crucial as the automotive industry continues to embrace innovative materials and production techniques, paving the way for more sustainable and effective vehicle manufacturing solutions.

In this context, the innovative dimple spot welding (DSW) method developed by Sakaguchi et al. [20] represents an interesting advancement. DSW leverages existing resistance spot welding infrastructure to join aluminium alloys and steel sheets efficiently. This method involves creating a dimple in the steel and drilling a corresponding hole in the aluminium, allowing for a secure mechanical interlock when welded. Preliminary tests indicate that DSW joints exhibit static and fatigue strengths comparable to those achieved by SPR, thus offering a promising solution for integrating lightweight materials into automotive structures without necessitating costly equipment modifications.

Under this scenario, the aim of this paper is to numerically analyse dimple spot welding by means of the implicit gradient approach [21,22,23]. The analyses are performed by considering the characteristic fatigue strength of welded steel and aluminium alloy. The fatigue behaviour of dimple spot welds is analysed by investigating various models that incorporate the contact interaction between the two plates.

## 2. Theoretical Background

This paper explores a sophisticated approach to addressing notch effects in fatigue analysis by defining an effective non-local stress that captures the stress field surrounding critical points in a material. The foundation of this approach lies in the non-local theory, which suggests that the stress at a point cannot be understood solely by local values but should also incorporate the influence of surrounding points.

To formalise this concept, the effective stress *σ_eff_*(*P*) at any point *P* within a volume *V* is defined as a weighted average of equivalent stress *σ_eq_*, represented mathematically as:

(1) $$\sigma_{eff}(P)=\frac{\int_{V}\Psi (P,Q)\sigma_{eq}(Q)dV}{\int_{V}\Psi (P,Q)dV}$$

where *Q* is a variable point in the volume *V*, and *Ψ* is an isotropic weight function that diminishes with increasing distance ∣*P* − *Q*∣. This function accounts for the stress gradient in the local field, enhancing the predictive capabilities of the model. The reference volume $\int_{V}\Psi (P,Q)dV$ normalises the weighted average, ensuring that the effective stress is dimensionally consistent.

According to Peerlings at al. [24], this integral definition of effective stress *σ_eff_*, can be approximated by transforming Equation (1) into an inhomogeneous Helmholtz equation:

(2) $$\sigma_{eff}-c^{2}\nabla^{2}\sigma_{eff}=\sigma_{eq}\;in\;V$$

where ∇^2^ denotes the Laplace operator, and *c* represents a characteristic length related to the material properties. This transformation is significant, as it allows the effective stress to be solved more easily than the original integral formulation given by Equation (1), particularly in three-dimensional domains where the critical point cannot be predetermined.

To apply this framework in practical scenarios, the authors consider Neumann-type boundary conditions, which facilitate the numerical solution of the Helmholtz equation. This strategy is further supported by the use of commercial finite element software (COMSOL Multiphysics^TM^), where second-order tetrahedral elements are employed to mesh the three-dimensional models.

Moreover, the paper cites early contributions to the non-local theory by Kroner, Eringen, and Edelen [25], which laid the groundwork for understanding stress as a function of averaged strain within a representative volume of material. This theoretical background is crucial for modelling materials that exhibit strain-softening behaviour, as proposed by Pijaudier-Cabot and Bazant [26,27].

The paper emphasises that under fatigue loading, stress variations are critical for defining equivalent stress. In the case with proportional and uniaxial loading scenarios, the maximum principal stress variation Δ*σ*_1_ can be effectively used to characterise the equivalent stress range (Δ*σ_eq_* = Δ*σ*_1_), leading to equations that streamline the analysis:

(3) $$\Delta \sigma_{eff}-c^{2}\nabla^{2}\;\Delta \sigma_{eff}=\Delta \sigma_{eq}$$

In particular, for welded joints, the characteristic length *c* is specified as 0.2 mm, whereas this becomes 0.15 for welded joints made of aluminium alloy.

Furthermore, the value of *c* can be calculated according to Equation (4) (see ref. [28]):

(4) $$c=za_{0}$$

where, for fatigue loading, the El-Haddad [29] critical length *a*_0_ takes the form:

(5) $$a_{0}=\frac{1}{\pi }{\left(\frac{\Delta K_{th}}{\Delta \sigma_{0}}\right)}^{2}$$

where Δ*σ*_0_ is the fatigue limit of the base material and Δ*K_th_* is the threshold range of the Stress Intensity Factor. The parameter *z* is a constant that depends on the equivalent stress *σ_eq_*. When the equivalent stress *σ_eq_* is assumed to be equal to the maximum principal stress, *z* becomes 0.545.

Equations (4) and (5) allow us to assess the value of *c* when the relevant material properties of the parent material and the cracked specimen are known.

**Figure 1:**
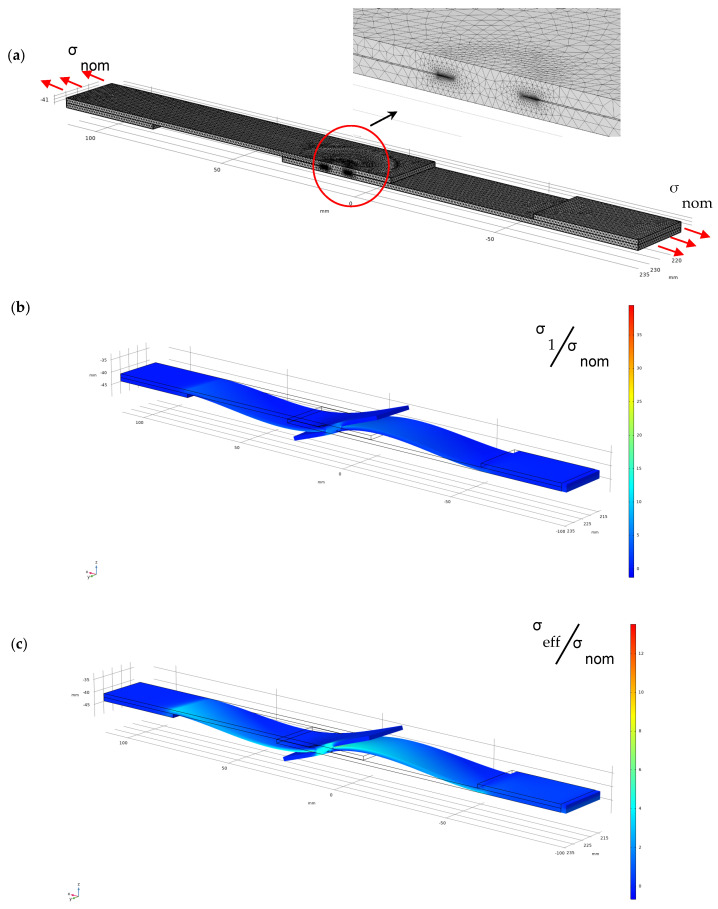
Effective stress *σ_eff_* for spot weld joint evaluated by solving Equation (2), assuming that *σ_eq_* coincides with the maximum principal stress *σ*_1_. The plate is subjected to a nominal tensile stress *σ_nom_*; (**a**) mesh; (**b**) maximum principal stress *σ*_1_; and (**c**) effective stress *σ_eff_*.

In Figure 1, typical spot weld joints subjected to tensile loadings are reported. Due to symmetry, only half the model is reported in the figure. The mesh is only fine near the weld where the stress becomes singular due to the sharp V-notch. The deformed shape in Figure 1b indicates that, at the two ends of the specimens, the rotation is neglected. The maximum principal stress tends to infinity at the notch tip whereas the effective stress *σ_eff_* obtained by solving Equation (2) at the weld is not singular and assumes a finite value [21,22,24,30]. Hence, the value of Δ*σ_eff_*, related to the range of nominal stress Δ*σ_nom_*, can be used to evaluate the fatigue life of components. The location at which the maximum value of Δ*σ_eff_* occurs will be the most likely site for fatigue nucleation.

**Figure 2:**
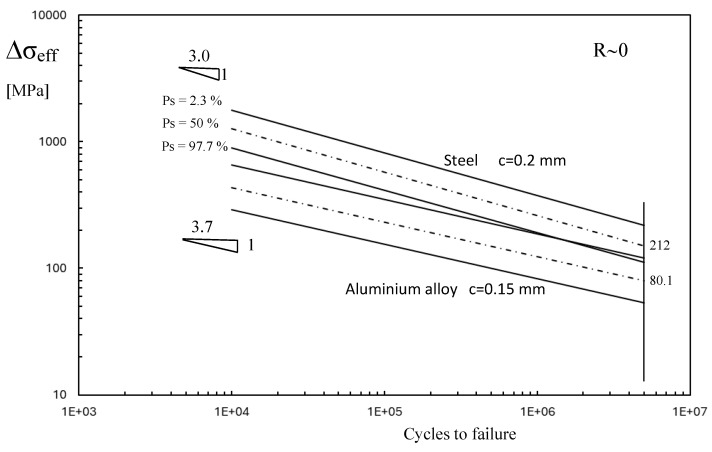
Scatter band of steel welded joints [21] in terms of maximum effective stress range (scatter bands related to mean values plus/minus 2 standard deviations; *Ps:* probability of survival).

Figure 2 shows the fatigue curve for welded joints obtained from about 600 experimental data taken from literature, mainly in relation to the fatigue strength of cruciform and T joints subjected to tensile or bending loading. The fatigue strength, in terms of the range of effective stress Δ*σ_eff_*, depends only on the material: steels or aluminium alloy. The two scatter bands are separated and present different slopes: 3.0 for the weld made of steel and 3.7 for the weld made of aluminium alloy. For a complex welded joint, by solving Equation (4), the maximum effective stress range Δ*σ_eff_* is the main physical parameter that, as introduced in Figure 2, gives us the fatigue life of components independently of the shape of the weld, and the size and type of loading (bending or tensile stress).

## 3. Dimple Spot Welding

This section summarises the interesting experimental work on a novel type of connection between dissimilar materials, such as steel and aluminium alloys, proposed by Sakaguchi et al. [20].

### 3.1. Geometry

Dimple spot welding, as proposed by Sakaguchi et al. [20], is a process that uses a spot weld from two overlapping metal pieces and locks the displacement of an interposed sheet. The process begins by forming a dimple in a steel sheet and drilling a hole in an aluminium alloy plate. A backing steel plate is then prepared. The aluminium plate is positioned between the steel sheet and the backing plate, with the dimple from the steel sheet aligning through the hole in the aluminium. Resistance spot welding is used to join the steel sheet and backing plate, securely clamping the aluminium plate in place. A key advantage of this dimple spot welding method is that it utilises existing resistance spot welding equipment, allowing for seamless integration into current production lines without requiring modifications.

**Figure 3:**
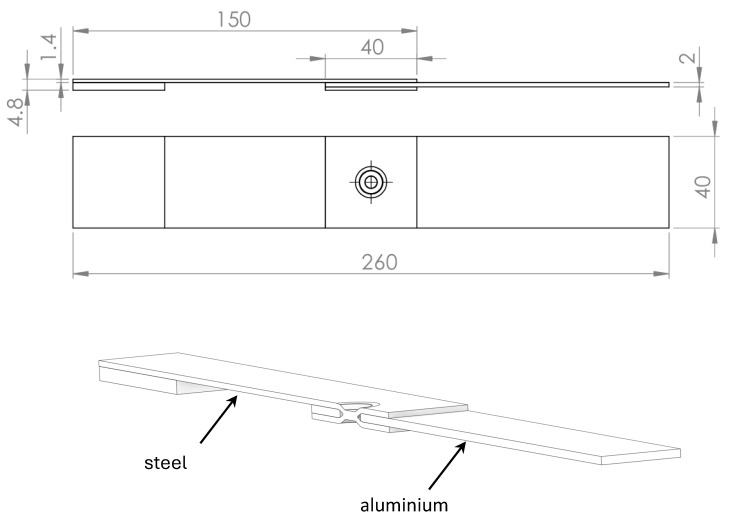
Specimen used in the experimental analysis [20].

Figure 3 reports the geometry of the dimple spot welding analysed in [20]. The thickness of the two joined plates is 1.4 mm and 2.0 mm for steel and for aluminium alloy, respectively. A tab is present to align the load when remote tensile loading is applied. The overlapping area is a square of 40 × 40 mm, and the spot weld has a diameter of 5.5 mm.

### 3.2. Materials

Table 1 shows the material used in the four series of specimens for the plate made of steel and aluminium alloy. Table 2 presents the mechanical properties of the materials.

In order to calculate the material parameter *c* for the steel weld, a common approach is to use the value of 0.2 mm for the arc welding process, independently of the material properties [30,31]. Unfortunately, for the aluminium plate, the fatigue limit and the fatigue life curve of the base material as well as the threshold range of the stress intensity factor are not reported in reference [20]. The exact values can vary depending on factors such as the material heat treatment, environmental conditions, and the specific testing method. If we estimate these two important strength parameters from the tensile strength reported in Table 2 or from the average value reported in the literature, the size of *c* estimated by means of Equations (4) and (5) is around some tenths of a millimetre. However, due to the inherent uncertainty in this calculation, the typical value for welded joints made of aluminium alloy will be utilised. This is a strong exemplification of the problem. However, in this case we know the fatigue curve of the material and the characteristic length *c* that can address some important general information about fatigue performance of dimple spot welding.

### 3.3. Fatigue Strength

**Figure 4:**
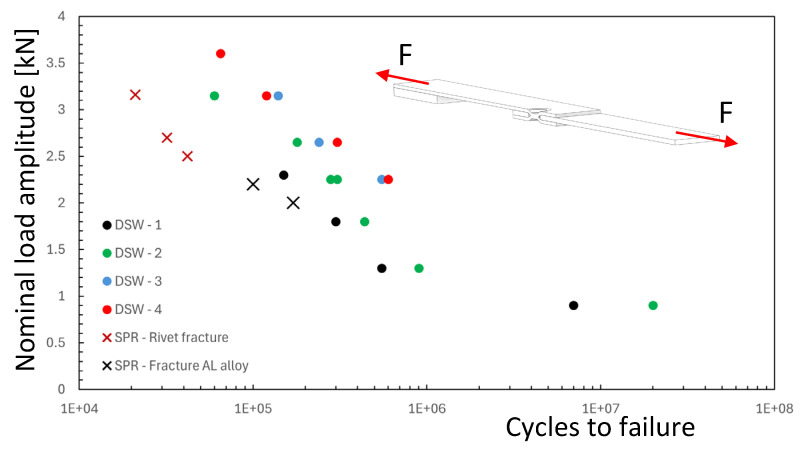
Comparison between the fatigue strength of dimple spot welding (DSW) joints and the self-piercing riveting (SPR) joints under tensile loading. Nominal load ratio R = 0.1 (F: nominal load; experimental results from reference [20]).

**Figure 5:**
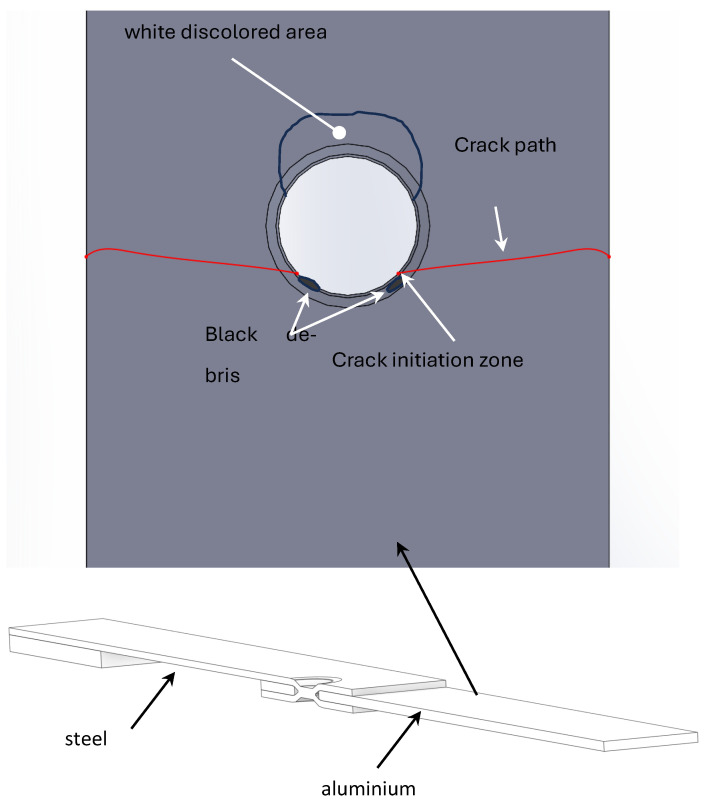
Typical fatigue failure at the aluminium plate.

Fatigue tests were conducted at room temperature at constant amplitude and the nominal load ratio *R* was set equal to 0.1 [20]. The fatigue life comparison between dimple spot welding and self-piercing riveting joints shows that the DSW specimens, made from the same materials (SPC980 steel and A7003 aluminium alloy), exhibit fatigue lives approximately 10 times longer than self-piercing riveting specimens. DSW joints demonstrated superior fatigue strength, with all failures occurring in the aluminium plate. Sakaguchi et al. [20] underline that the fatigue life of DSW specimens depends on both the aluminium alloy strength and the steel sheet strength, with higher-strength materials increasing fatigue life. Figure 4 reports the comparison, in terms of fatigue strength, between the nominal stress applied to DSW specimens and that of SPR joints, which will be used in the next section for the numerical simulation. After failure, the analysis of the aluminium plate of DSW specimens shows a white discoloured area due to the repeated contact and friction observed at the top side while the crack nucleates in the opposite side of the hole where black debris was observed, as indicated in Figure 5. Sakaguchi et al. [20] justified this behaviour by observing that, in their FE analysis, the higher tangential stress area corresponded well to the actual site of crack initiation.

## 4. Numerical Analysis of Dimple Spot Welding

### 4.1. FE Model

In order to simulate, with a tree-dimensional approach, the fatigue behaviour of dimple spot welding joints, a detailed FE analysis will be performed by means of two pieces of software: Ansys Workbench and Comsol Multiphysics. This is necessary because the complicated task of simulating the contact of the sheets, as reported in Figure 3, with or without the friction coefficients, will be undertaken using the Ansys code. Then, the FE results, such as the maximum principal stress, will be exported and used by Comsol Multiphysics as input data to solve the Helmholtz Equations (2) or (3).

**Figure 6:**
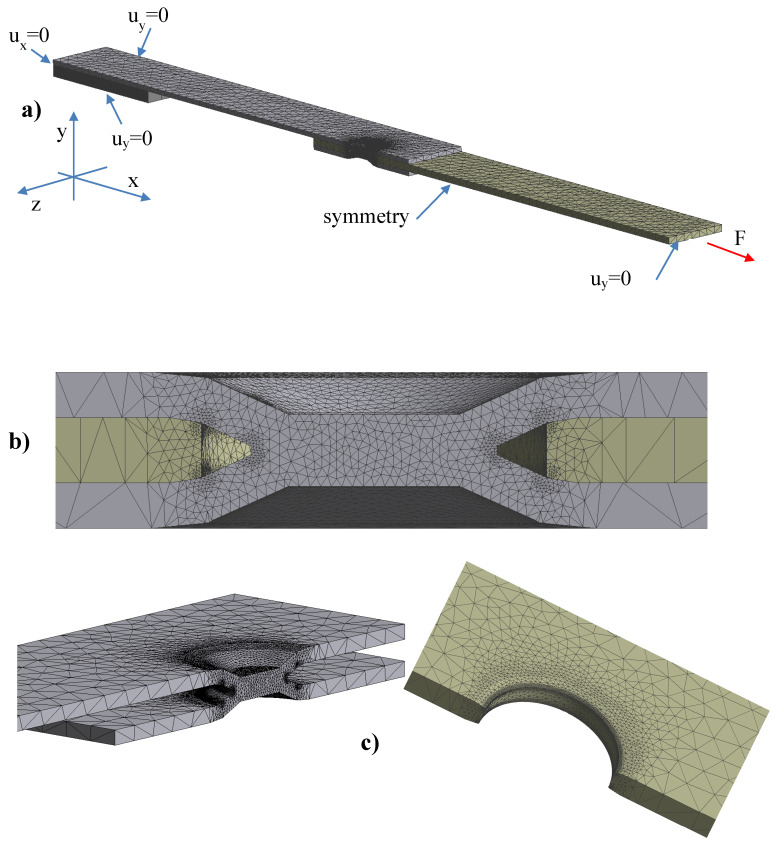
FE model and boundary conditions for the dimple spot welding; (**a**) Boundary conditions used in the model (u: displacement); (**b**) Mesh detail around the spot weld; (**c**) Mesh for spot weld and aluminium plate around the hole.

The three-dimensional model was built by means of 3D Cad and reproduces the details of the geometry around the spot weld, as appears in reference [20]. Figure 6 shows the detail of the FE mesh used for the DSW joints. Due to the symmetry, only half the joint was modelled. At both ends, rotations were prevented by setting as zero the displacement perpendicular to the longitudinal plane (*u_y_* in Figure 6a) and a force was applied at one end while restraining longitudinal displacements at the other (*u_x_* in Figure 6a). Figure 6b shows the details of the mesh used in the FE analysis. Tetragonal elements were used with quadratic element order in the Ansys software, while in Comsol second-order element with Lagrange shape function was adopted. The size of the smallest element was in the order of 10^−1^ mm at the weld for the steel plate and all around the hole of the aluminium plate. A convergence analysis was performed to obtain a stable value of effective stress in the contact zone. Despite an oscillation value of the maximum principal stress in the zone where the plates are in contact (around the white discoloured area in Figure 5), the value of effective stress also resulted stable in that area. This is an advantage of the numerical implicit technique adopted in this study. The use of more accurate meshes would result in excessively long computation times. On the other hand, due to the uncertainty associated with the characteristic length *c*, the accuracy in the fatigue life prediction can only be accurate in the zone around the spot weld (see nest sections). As a general rule, convergence in finite element analysis is achieved when the minimum size of the elements in the region of highest stress is in the same order of magnitude as the characteristic length *c* [30].

The residual stress due to the weld process was not considered in this study when no stress release was performed [31].

The material was considered as linear elastic. The Poisson’s ratios of the steel sheets and aluminium alloys were set to 0.30 and 0.34, respectively. The Young modulus of 200 GPa was considered for the steel plate and was reduced to 70 GPa for the aluminium plate.

### 4.2. Results with Frictionless Simulation

**Figure 7:**
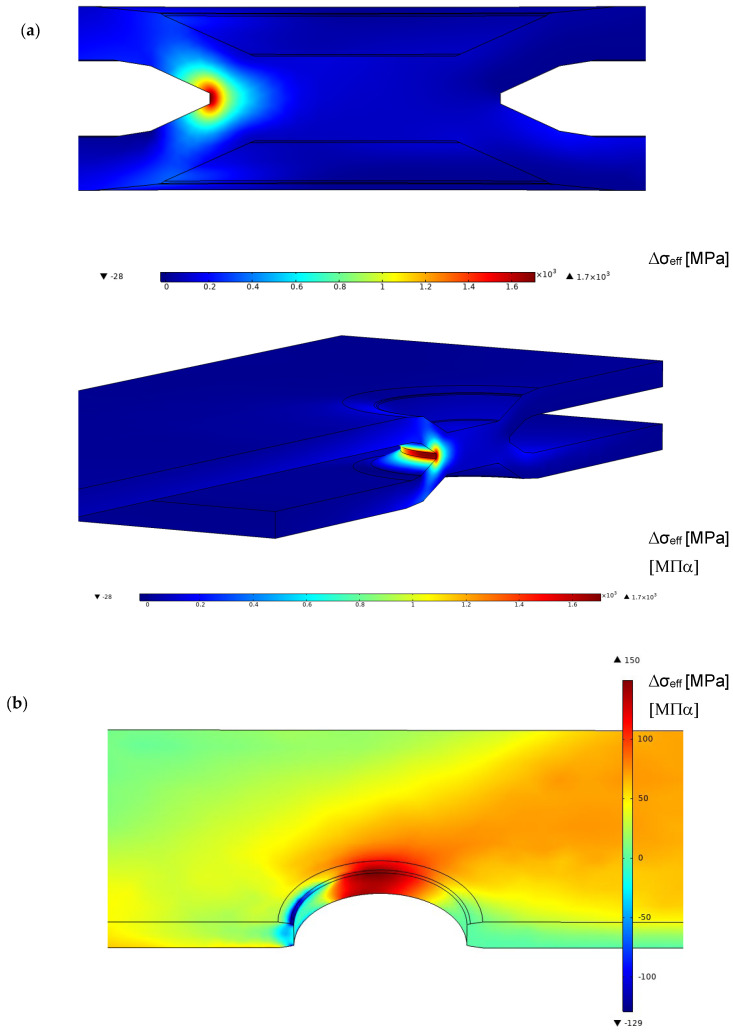
Range of effective stress in the dimple spot welding around the spot weld and the toe for a remote applied force range of 4.5 kN for the frictionless model (**a**) steel plate; (**b**) aluminium plate.

The first analysis was performed without taking into account the friction coefficient between the two plates. The load was transmitted by the wedge effect between the steel and the aluminium plate. Figure 7 reports the values of the range of effective stress related to a load range of 4.5 kN applied at the remote section. This load, applied during the dimple spot welding, involves an average fatigue life of around 3·× 10^5^ cycles, as appears in Figure 4 for DSW2. The results reported in Figure 7, as well as other similar results reported in the figure below, are focalised around the spot weld because it is the place of highest stress level for both the aluminium and the steel plate. For the steel plate, the maximum effective stress range is at the weld, whereas for the aluminium plate, the maximum is localised at the central part of the hole. Furthermore, the FE simulation results indicate that the contact zone between the two plates is localised around the white discoloured area observed in Figure 5.

**Figure 8:**
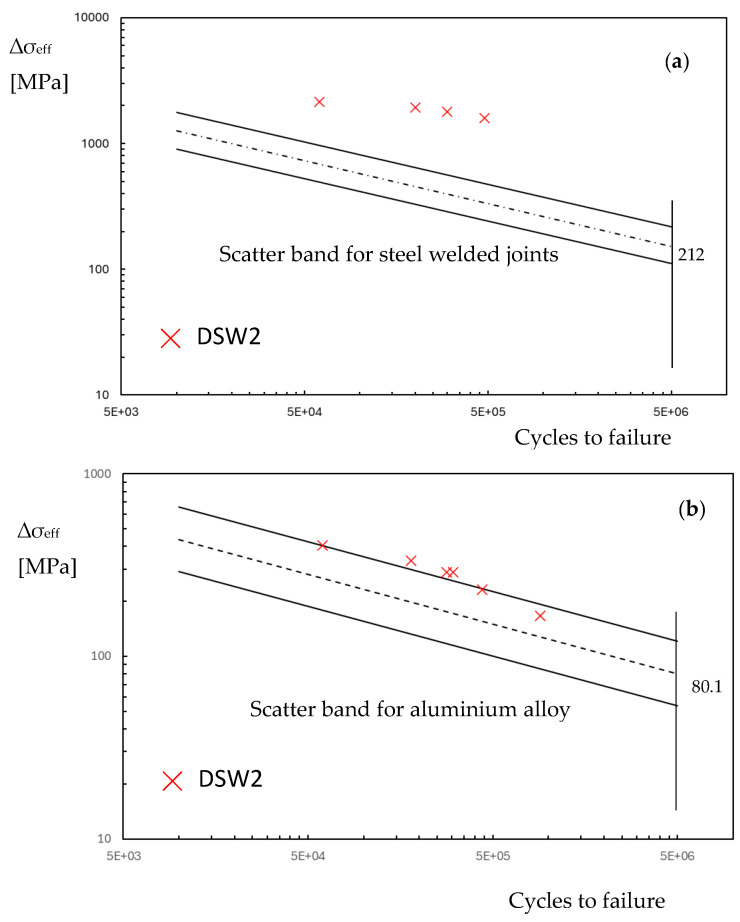
Fatigue life in terms of effective stress range in the dimple spot welding for the FE frictionless model (**a**) steel plate at the weld; (**b**) aluminium plate at the hole.

By considering series DSW2 as a reference, Figure 8 reports the experimental points in terms of effective stress range over the fatigue diagram of Figure 2. The experimental points of the aluminium plate fall into the scatter band of the aluminium alloy weld, but the steel spot weld shows a very high stress level. If this were true, the failure should occur in the steel plate and a fatigue crack should be visible at the weld, but this is in contrast with the experiments.

For the aluminium plate, the maximum effective stress, as shown in Figure 7, does not exactly agree with the points where the fatigue crack nucleates. Furthermore, the value of the characteristic length *c* and the Woehler curve of the aluminium plate in Table 2 are not known, and were approximated with those of the welded aluminium alloy. Nevertheless, in the spirit of a simplified analysis, the experimental points are located on the upper part of the welded aluminium alloy scatter band.

### 4.3. Results with Friction Simulation

**Figure 9:**
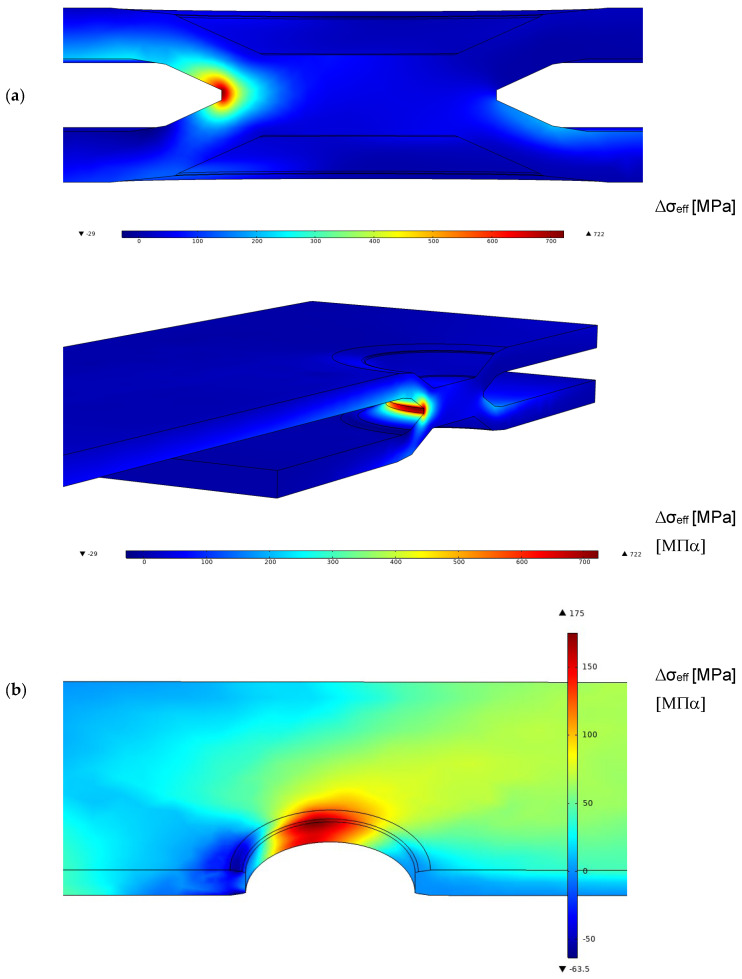
Range of effective stress in the dimple spot welding around the spot weld and the weld toe for a remote applied force range of 4.5 kN for the model with a friction coefficient of 0.45 (**a**) steel plate; (**b**) aluminium plate.

In this section, a friction coefficient between the two plates of Figure 3 was introduced. Sakaguchi at al. [20] discussed the rule of friction coefficients ranging between 0.38 and 0.56, but in our simulations a reference value of 0.45 was considered independently of the couple of plates used (see Table 1 for the friction coefficients of each series). This constitutes a simplification of the contact problem, as a more accurate model would account for the influence of relative speed, surface topography, and contact pressures [32,33,34]. Figure 9 reports the effective stress related to a load range of 4.5 kN applied at the remote section, as shown in Figure 7. The trend of effective stress is similar to the case of the frictionless model but with lower values in the steel plate and higher values in the aluminium plate.

**Figure 10:**
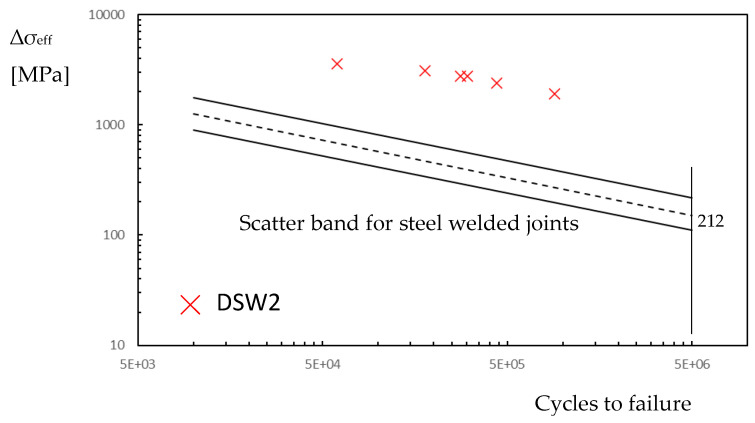
Fatigue life in terms of effective stress range in the dimple spot welding for the FE friction model (steel plate at the weld).

Similar to the frictionless simulation, assuming that the effective stress range corresponds to that of monotonic loading, it is possible to plot the experimental points of series DSW2 on the fatigue diagram in Figure 2 by considering the friction coefficient. The introduction of the friction coefficient does not substantially modify the fatigue behaviour of the steel plate, as reported in Figure 10.

### 4.4. Results with Friction Simulation and Fatigue Loading Simulation

**Figure 11:**
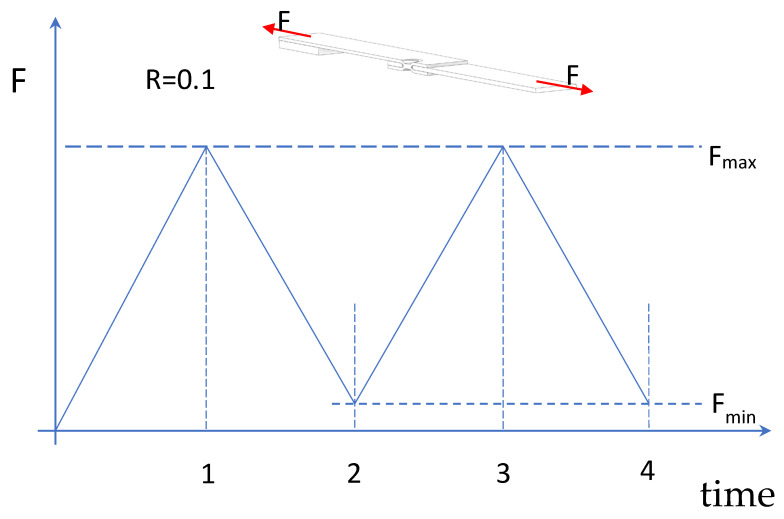
Load history at the nominal section for a nominal load ratio *R* of 0.1.

In this section, a friction coefficient of 0.45 was incorporated into the model, as in the previous section. However, the initial load cycles were simulated following the approach outlined in a previous study that numerically investigated the fatigue behaviour of double-riveted lap joints [35]. In reference [32], it was verified that the effect of the friction force tends to reduce the range of stresses at the net section during fatigue loadings. Therefore, in this study, the nominal load was considered as variable, as reported in Figure 11. Two complete cycles were simulated. The results at times 1 and 3 are quite similar, as are those at times 2 and 4, indicating that simulating additional cycles is unlikely to produce significant changes.

**Figure 12:**
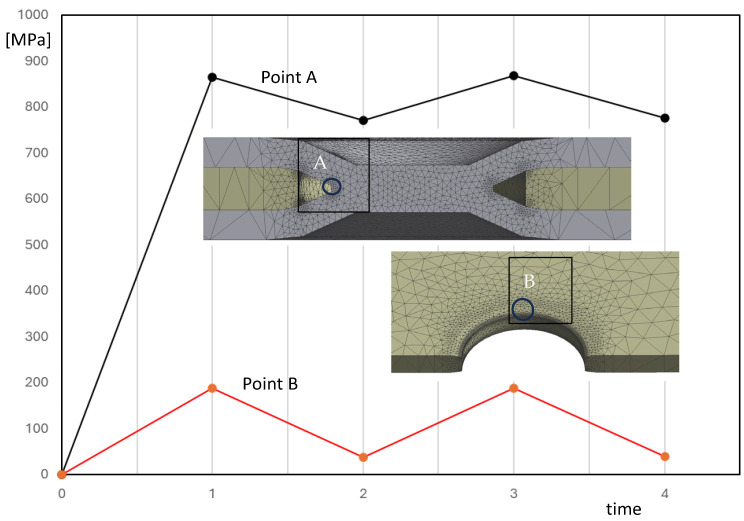
Effective stress range versus time at the weld (point A, steel plate) and at the border of the aluminium plate (point B) for the model with a friction coefficient of 0.45 by simulating the fatigue loading (ΔF = 2250 N, F_max_ = 2500 N, F_min_ = 250 N, nominal load ratio R = 0.1).

Figure 12 reveals that, despite a nominal load ratio R of 0.1, the effective stress range at point A is significantly lower than expected. In a frictionless scenario, the effective stress range is 90% of the maximum stress level. This discrepancy is less pronounced at point B in the aluminium plate.

**Figure 13:**
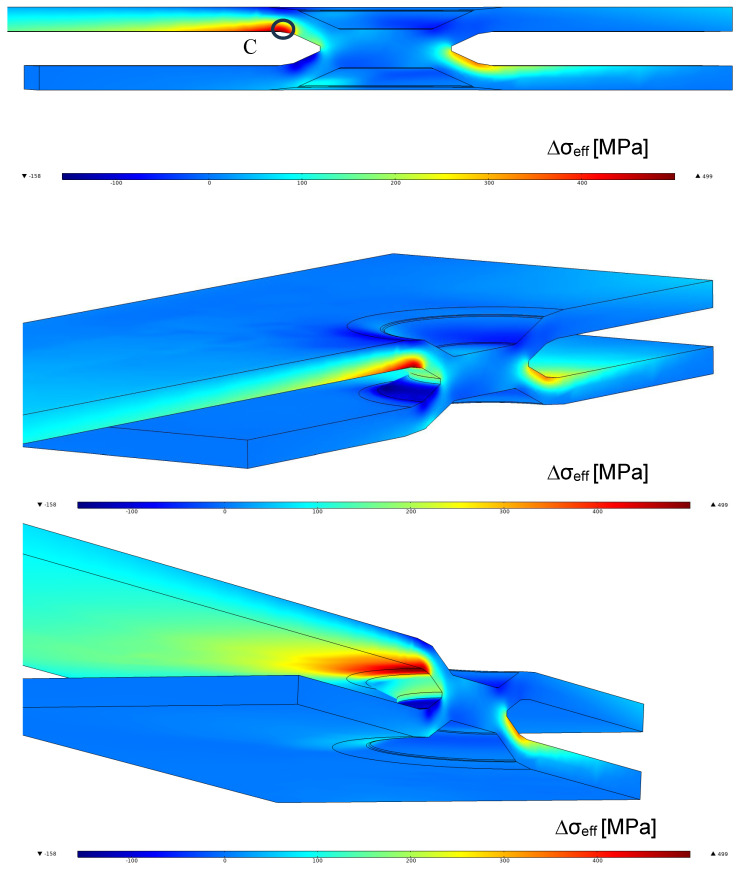
Range of effective stress in the dimple spot welding around the spot weld and the weld toe for a remote applied force range of ΔF = 4.5 kN (F_max_ = 5 kN, F_min_ = 500 N, nominal load ratio R = 0.1) for the model with a friction coefficient of 0.45 by simulating the fatigue loading (steel plate).

**Figure 14:**
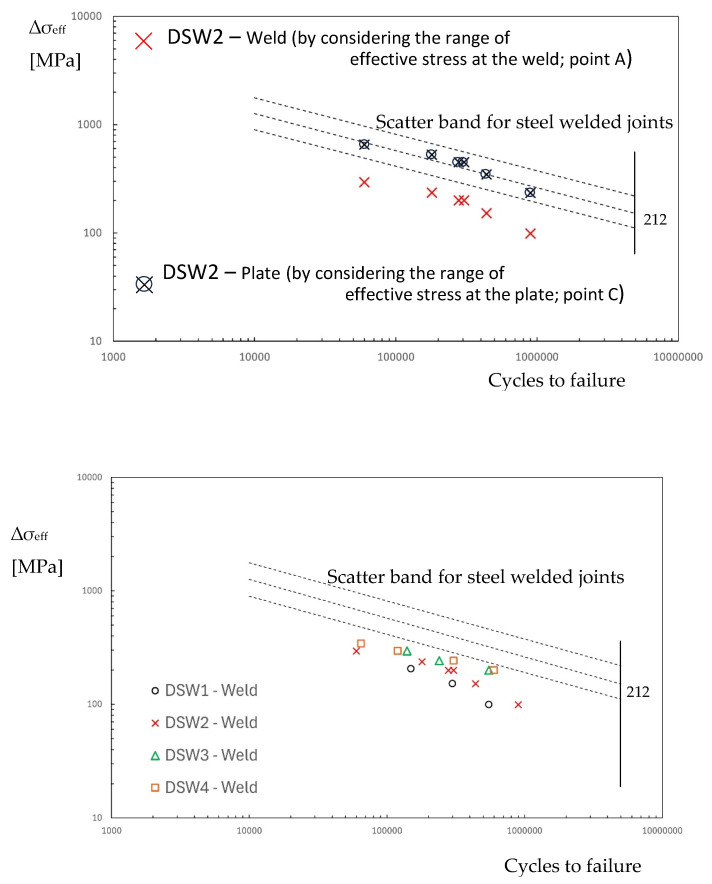
Fatigue life in terms of effective stress range in the dimple spot welding for the FE with friction simulation and fatigue lading simulation (steel plate).

In order to obtain the fatigue damage of the weld, without taking into account a multiaxial fatigue criterion [36], at any points of the dimple spot welding, the difference between the maximum principal stress at time 3 and the maximum principal stress at time 4 is considered. Now, by integrating Equation (3), we obtain the range of effective stress to introduce in the fatigue curve of Figure 2. Figure 13 shows the range of effective stress due to the range of principal stress. The position of maximum effective stress moves from the weld to point *C*. In this region, the characteristic length may differ from that of the welded zone. However, due to the lack of precise knowledge regarding the correct value of *c* for the steel plate, and for the sake of simplicity, the fatigue resistance of welded steel structures is assumed to be equal to that of the weld throughout the plate. Figure 14 reports the fatigue points in terms of the effective stress range at the weld (point A) or at the plate (point *C*). In Figure 14, the points relative to *C* fall inside the scatter band but are not at all significative because of the uncertainty in the fatigue properties. On the contrary, the experimental points related to the fatigue strength of the weld show a low value of effective stress range, and this respects the condition that the fatigue failure does not occur in the steel plate. Finally, Figure 14 also includes the experimental data points for the other series, considering the effective stress at the weld toe.

## 5. Conclusions

This paper analysed the stress distribution and fatigue life under cyclic loading of dimple spot weld (DSW) used to connect two different plates made of steel and aluminium alloy, respectively.

The frictionless model results in unusually high stress on the steel plate, which deviates from experimental observations showing that fatigue cracks initiate in the aluminium plate. This discrepancy implies that, in practical DSW applications, additional factors such as friction likely play a role in altering stress distribution and crack locations.

By adding a friction coefficient in the FE model with a monotonic loading, the analysis reveals a decrease in stress concentration at the weld, but around the aluminium plate hole the magnitude of the effective peak stress increases.

Through cyclic loading simulations, the fatigue analysis underscores the critical importance of both friction and load history in accurately predicting fatigue life. Friction significantly reduces the range of effective stress at the weld.

To enhance the accuracy of numerical predictions in future work, incorporating a non-linear material model, such as elasto-plastic, and a more detailed friction model, should be considered.

## Figures and Tables

**Table 1:** Steel sheets and aluminium alloys considered in the dimple spot welding [20].

Specimen	Aluminium Alloy	Steel Sheet	Friction Coefficient
DSW-1	A6N01	SPC590	0.56
DSW-2	A7003	SPC590	0.45
DSW-3	A6N01	SPC980	0.42
DSW-4	A7003	SPC980	0.38

**Table 2:** Ultimate tensile strength, yield strength, and elongation of material used in dimple spot welding [20].

Material	Ultimate Tensile Strength (MPa)	Yield Strength (MPa)	Elongation (%)
A6N01	257	225	9.4
A7003	395	349	10.8
SPC590	590	444	24.8
SPC980	980	813	13.9

## Data Availability

The original contributions presented in this study are included in the article. Further inquiries can be directed to the corresponding author.
